# A Protein Domain and Family Based Approach to Rare Variant Association Analysis

**DOI:** 10.1371/journal.pone.0153803

**Published:** 2016-04-29

**Authors:** Tom G. Richardson, Hashem A. Shihab, Manuel A. Rivas, Mark I. McCarthy, Colin Campbell, Nicholas J. Timpson, Tom R. Gaunt

**Affiliations:** 1 MRC Integrative Epidemiology Unit, School of Social and Community Medicine, University of Bristol, Bristol, United Kingdom; 2 Wellcome Trust Centre for Human Genetics, Nuffield Department of Clinical Medicine, University of Oxford, Oxford, United Kingdom; 3 Oxford Centre for Diabetes Endocrinology and Metabolism, University of Oxford, Oxford, United Kingdom; 4 Intelligent Systems Laboratory, University of Bristol, Bristol, United Kingdom; UCL Institute of Neurology, UNITED KINGDOM

## Abstract

**Background:**

It has become common practice to analyse large scale sequencing data with statistical approaches based around the aggregation of rare variants within the same gene. We applied a novel approach to rare variant analysis by collapsing variants together using protein domain and family coordinates, regarded to be a more discrete definition of a biologically functional unit.

**Methods:**

Using Pfam definitions, we collapsed rare variants (Minor Allele Frequency ≤ 1%) together in three different ways 1) variants within single genomic regions which map to individual protein domains 2) variants within two individual protein domain regions which are predicted to be responsible for a protein-protein interaction 3) all variants within combined regions from multiple genes responsible for coding the same protein domain (i.e. protein families). A conventional collapsing analysis using gene coordinates was also undertaken for comparison. We used UK10K sequence data and investigated associations between regions of variants and lipid traits using the sequence kernel association test (SKAT).

**Results:**

We observed no strong evidence of association between regions of variants based on Pfam domain definitions and lipid traits. Quantile-Quantile plots illustrated that the overall distributions of p-values from the protein domain analyses were comparable to that of a conventional gene-based approach. Deviations from this distribution suggested that collapsing by either protein domain or gene definitions may be favourable depending on the trait analysed.

**Conclusion:**

We have collapsed rare variants together using protein domain and family coordinates to present an alternative approach over collapsing across conventionally used gene-based regions. Although no strong evidence of association was detected in these analyses, future studies may still find value in adopting these approaches to detect previously unidentified association signals.

## Introduction

Despite the success in identifying genetic associations with complex disease in recent years, we are still relatively unaware of the proportion of this phenotypic variation that rare variants are responsible for. Study designs over the last decade have largely concerned common variants, and whilst the amount of additive genetic variance explained by these variants is greater than initially expected[1], the case of the “missing heritability” still remains. Endeavors have therefore shifted to uncover the role of rare variants, with potentially much larger effect sizes than those observed from common variants[2, 3]. Due to breakthroughs in next generation sequencing we now have a wealth of data consisting of rare variants, paving the way for the development of novel methodology that allows us to investigate the impact of rare genetic variation on complex disease[4]. These methods should become particularly useful once next generation sequencing becomes more extensively undertaken in large population collections.

One type of approach involves grouping all variants within the same gene together, as they are likely to mark functional effects on the same protein or RNA, followed by analysing the combined effect of these variants using recently developed association tests. However, typically these approaches are underpowered[5]. A major cause of this is due to collapsing variants with contrasting directions of effect, as well as variants with little to no effect (neutral variants), which has inspired the development of variance-component tests (e.g. C-Alpha[6], SKAT[7]). Another plausible explanation for this could be that variants within the same genomic region that are grouped together do not necessarily share similar function. Analyses may therefore benefit from redefining the region of interest on the basis of likely functional consequences. Doing so may identify a more unified potential effect from sets of variants, not observed when collapsing across entire genes.

Protein domains are distinct functional, structural and evolutionary units which can either span sub-sections of a protein or its entire length. The exploration of human disease mutations have found that they tend to cluster together within highly conserved protein positions[8, 9], which certain protein domains occupy[10]. Amongst other functional tasks, protein domains can interact with each other and lead to protein-protein interactions (PPIs). It is understood that mutations that affect the binding interface of proteins can lead to dysfunctional allosteric changes which can have a downstream effect on disease[11]. Domains which consist of the same DNA sequence may occur multiple times across the genome in different genomic regions but can have a similar functional consequence. These regions are collectively referred to as protein families.

In comparison to conventional approaches which aggregate variants according to gene coordinates, we have undertaken a study to evaluate whether collapsing variants across regions based on protein domain coordinates provides a viable alternative. We aggregated variants together using 3 different definitions:

1. **Protein domains:** Individual regions of the genome which mapped to individual protein domains. Certain protein domains can consist of the entire protein, which would therefore result in an identical analysis to using gene coordinates when analysing variants in exons. The exception to this would be variants at the 3’ and 5’ locations. However, protein domains can also consist of subsections of the protein, which would therefore only map back to a subsection of the corresponding gene region. These individual regions therefore varied in length based on definitions. In these instances we hypothesised that using domain coordinates may aggregate together more functionally relevant variants, thus resulting in a gain of statistical power in the resulting analysis.
2. **Domain-Domain Interactions:** As two individual protein domains can interact and subsequently lead to the formation of PPIs, it is plausible that the corresponding pair of genomic regions may harbour functionally relevant variants. Whilst a mutation in either domain may be sufficient to affect binding affinity, analysing these regions together may provide stronger evidence of association than analysing each individually as they affect the same interaction. Consequently this definition consists of all variants from within two of the regions analysed using the previous definition, which were predicted to be responsible for a PPI.
3. **Protein Families:** As previously mentioned, protein families can be defined as multiple protein domains which have a similar sequence and structure. This final definition therefore consists of multiple genomic regions which map to the same type of protein domain. Certain protein families can consist of a large frequency of domains and in these circumstances it seemed unlikely that so many different proteins would be involved along the causal pathway of disease. However, as these protein domains can have similar functionality it was worthwhile investigating whether variants within these regions, after selecting only genes with experimental evidence of interaction, were collectively associated with disease.

We hypothesised that, in comparison to conventionally used gene coordinates, aggregating rare variants together across these alternative definitions may result in a greater proportion of variants which have a similar impact along the causal pathway and fewer neutral variants which dilute the observed signal. The potential trade-off to this is that variants with a similar functional effect within the same gene region may end up being collapsed separately. Rare variant analyses using these alternative definitions were conducted using samples from individuals involved in the UK10K project, which consists of participants drawn from the ALSPAC (Avon Longitudinal Study of Parents and Children) and TwinsUK cohorts.

## Methods

### Cohort Description

The UK10K consortium has two main project arms. In this study, we have used data from the cohorts’ arm which was designed to investigate the contribution of genome wide genetic variation to a range of quantitative traits. This arm contains individuals from two intensively studied cohorts of European ancestry, ALSPAC (Avon Longitudinal Study of Parents and Children) and TwinsUK:

#### ALSPAC

ALSPAC is a population-based cohort study investigating genetic and environmental factors that affect the health and development of children. The study methods are described in detail elsewhere[12, 13] (http://www.bristol.ac.uk/alspac).

Ethical approval was obtained from the National Research Ethics Service (NRES) Committee, South East London, REC 2. Written informed consent was obtained from parents for all measurements made.

#### TwinsUK

The TwinsUK registry is a cohort of volunteer adult twins from all over the United Kingdom[14]. Initially, only middle-aged women were recruited and as a result 83% of the registry is female. The registry currently contains 51% monozygotic (MZ) and 49% dizygotic (DZ) twins aged 18–103 years. Further details are available online (http://www.twinsuk.ac.uk/).

Informed consent was obtained from participants before they entered the study and ethical approval was granted by the National Research Ethics Service (NRES) Committee, Westminster, London.

### Sequencing Data

DNA Samples from 4,030 UK10K study participants (2,040 offspring from the ALSPAC cohort, 1,990 from the TwinsUK cohort) were subjected to low coverage (6-8x average read depth) whole-genome sequencing (WGS). Sequencing was performed at both the Wellcome Trust Sanger Institute (WTSI) and the Beijing Genomics Institute (BGI). DNA (1–3μg) was sheared to 100–1000 bp using a Covaris E210 or LE220 (Covaris, Woburn, MA, USA). Sheared DNA was size subjected to Illumina paired-end DNA library preparation. Following size selection (300–500 bp insert size), DNA libraries were sequenced using the Illumina HiSeq platform as paired-end 100 base reads according to manufacturer’s protocol.

Data that passed quality control (QC) was aligned to the GRCh37 human reference used in phase 1 of the 1000 Genomes Project. Reads were aligned using BWA (v0.5.9-r16)[15]. Of the 4,030 participants, 3,910 samples (1,976 ALSPAC and 1,934 TwinsUK) went through the variant calling procedure. Low quality samples were identified by comparing the samples to their GWAS genotypes using about 20,000 sites on chromosome 20. A total of 112 samples (48 ALSPAC and 64 TwinsUK) were removed, leaving 3,798 samples (1,928 ALSPAC and 1,870 TwinsUK) that were eligible for the genotype refinement phase.

Missing and low-confidence genotypes in the filtered VCFs were refined out using the imputation procedure in BEAGLE 4[16] with default parameters. Additional sample-level QC steps were carried out on refined genotypes, resulting in 17 samples (16 TwinsUK and 1 ALSPAC) being removed due to either non-reference discordance with GWAS SNV data>5%, multiple relations to other samples or failed sex check. A principal components analysis was conducted using EIGENSTRAT[17] to exclude participants of non-European ancestry after merging our data with a pruned 11 HapMap3 population dataset[18]. 44 subjects (12 TwinsUK and 32 ALSPAC) did not cluster to the European (CEU) cluster and were removed. The final sample size for association analyses comprised of 3,621 individuals which did not include any related pairs (1,754 TwinsUK and 1,867 ALSPAC).

### Data Collection

#### UK10K Phenotypes

**ALSPAC:** Non-fasting blood samples were taken from participants who attended the age 9 clinic (mean age: 9.9, range: 8.9–11.5). Plasma lipid concentrations (total cholesterol (TC), triglycerides (TG) and high density lipoprotein cholesterol (HDLc)) were measured by modification of the standard Lipid Research Clinics Protocol with enzymatic reagents for lipid determination[19]. Low density lipoprotein cholesterol (LDLc) concentration was subsequently calculated using the Friedwald equation[20]:

LDLc=TC–(HDLc+TG×0.45)

**TwinsUK:** Blood samples were taken after at least 6 hours of overnight fasting. The samples were immediately inverted three times and left to rest for 40 minutes at 4°C to obtain complete coagulation. The samples were then centrifuged for 10 min at 2000g and serum was removed. Four aliquots of 1.5 ml were placed into skirted micro centrifuge tubes and then stored in a -45°C freezer until sampling[21]. A colorimetric enzymatic method was used to determine TC, TG and HDL-c levels. The Friedewald equation was used to calculate LDL-c levels in subjects.

### Statistical Analysis

#### Simulated Data Analysis

As a proof of concept, we first undertook analyses using simulated data consisting of 3,000 individuals each with 5,000 rare variants (i.e. MAF ≤ 1%). 4,000 of these were neutral variants and 1,000 were disease causing variants (i.e. variants that were associated with the dichotomous phenotype). The odds ratios for the disease causing variants varied from 1.20 to 1.50, whereas the neutral variants only ever contributed to an odds ratio of 1.0. We randomly allocated variants into 250 ‘synthetic’ genes (i.e. not based on any genomic location, simply blocks of variants) of varying number of variants per gene (from 10 to 30 variants). Within these genes we defined subgroups of variants to resemble domains, varying in length between 5–20 variants. 1,000 of these synthetic domains were randomly selected for analysis. We conducted gene-based and domain-based analysis using the sequence kernel association test (SKAT)[7] to examine association between groups of variants within these regions and the synthetic phenotype. All simulated data was generated using PLINK v1.9[22].

#### Application to real data

**Fig 1:**
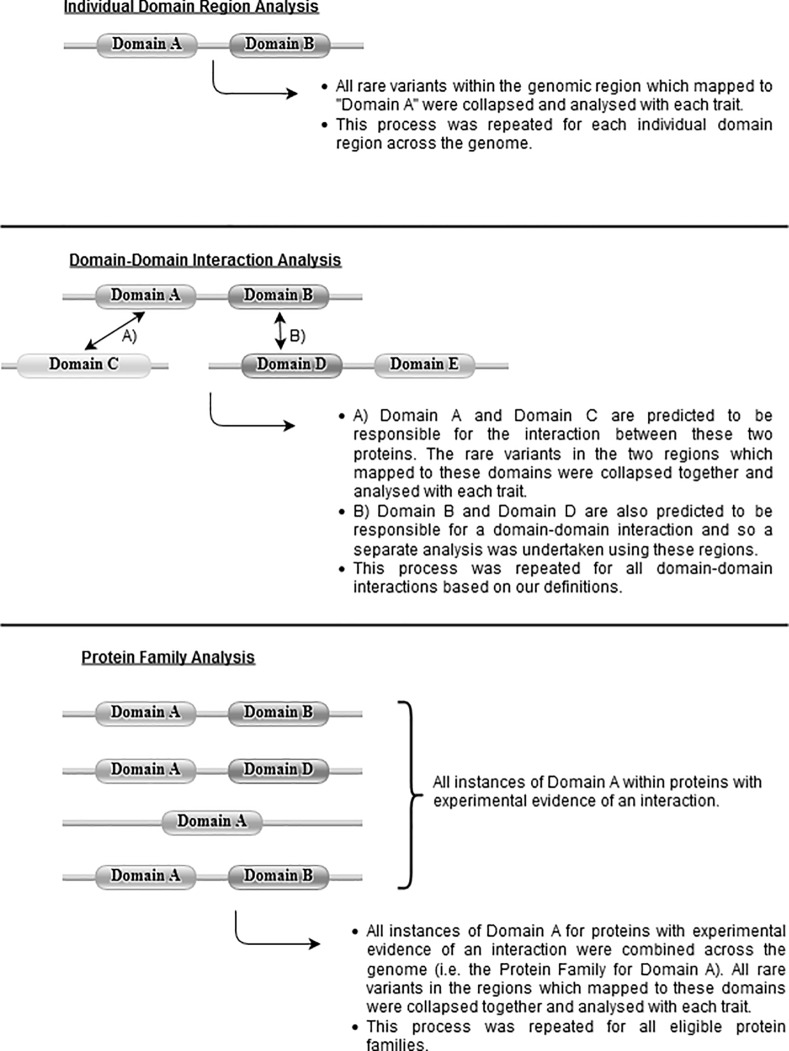
Graphical summary of how rare variants within domain regions were collapsed together for analysis. This figure describes how regions of rare variants were collapsed together and analysed: • Individual Domains: Regions which mapped to individual domains of a protein were analysed as opposed to the conventional approach of using individual gene regions. • Domain-Domain Interactions: Pairwise regions which mapped to two domains predicted to be responsible for a domain-domain interaction were analysed together. • Protein Families: All regions which mapped to the same type of protein domain within proteins which had experimental evidence of interaction were combined and analysed together. Domain images were created using the generate graphics feature from Pfam (located at http://pfam.xfam.org/generate_graphic).

Fig 1 summarises the planned analyses of this study. Using the UK10K sequence data, we took all Pfam protein family and domain coordinates and mapped them back to genomic coordinates using a custom Python script. These coordinates were extracted from the hg19 download used by the prediction tool Mutation Assessor [23]. Variants were filtered to only include those with a MAF≤1% and a CADD (Combined Annotation Dependent Depletion) C-Score ≥ 15. This threshold is suggested by the authors of CADD as it equates to the 5% most deleterious variants across the genome as predicted by this resource.

We conducted analyses on regions based on three novel definitions of a functional unit. These were:

1. **Protein domains:** Individual genomic regions which mapped to individual protein domains according to Pfam definitions. These regions comprised of either sections of proteins or in some cases the whole protein. For the latter, analyses would have been identical to analysing variants within the entire exonic region of the gene with the exception of variants at the 3’ and 5’ locations.
2. **Domain-Domain Interactions:** iPfam was used to identify which pairwise domains were predicted to be responsible for a given protein-protein interaction according to STRINGdb v9.1 [24]. Firstly, all pairwise protein interactions which had at least some experimental evidence and with a STRING score ≥ 0.8 were extracted. Then for each pairwise interaction it was verified whether any domains from the first protein interacted with any domains in the second protein, according to iPfam. If this was true, the two domain regions in questions were added to the list of eligible domain-domain interactions (DDI) for our analysis. All variants within regions that were responsible for each domain-domain interaction were aggregated and analysed together (i.e. all variants within two of the regions in the previous analysis which were predicted to be involved in a PPI).
3. **Protein Families:** Variants were collapsed together across regions that were located within the same type of domain across the genome (i.e. variants within all regions which had the same Pfam ID). However, only domains within gene regions whose product had experimental evidence of interaction according to STRINGdb (again using a STRING score ≥ 0.8 threshold) were combined. This meant that variants within multiple regions involved in the initial analysis (i.e. the individual domain analysis) were analysed together here.

Only regions which had at least 2 remaining variants were analysed using SKAT with each lipid trait (HDL, LDL, TC and TG) in turn. All traits were inverse normal transformed prior to analysis. Further details on trait standardization can be found in the Supplementary Material (S1 File).

To evaluate whether results provided strong evidence of association we used a threshold for multiple comparisons using the Bonferroni correction (i.e. 0.05/number of regions analysed). All individual protein domains regions were reanalysed using the SKAT-O test [25] found to have more power than SKAT in situations where variants within a region have the same direction of effect [26]. A single variant analysis was also conducted using each lipid trait for all rare variants which were analysed previously. This was to ensure that aggregating variants together across regions was not causing evidence of association observed from a single variant analysis to consequently become undetected. These results were plotted using Quantile-Quantile (Q-Q) plots.

#### Comparison between Individual Domain and Gene-based Results

Q-Q plots were generated using the distribution of p-values from the results of the individual protein domain analysis with results from a conventional gene-based analysis. This analysis was undertaken with the same dataset but using gene start and end coordinates according to hd19 definitions and analysed as before using SKAT with each lipid trait. The quantiles from the domain-based analyses were therefore interpolated as there were more individual domains analysed compared to the number of genes[27]. Q-Q plots were generated using the R package ‘qqman’[28]. All statistical analyses were undertaken using R statistical software[29].

## Results

### Simulated Data analysis

Analysing groups of variants from within simulated domain regions provided stronger evidence of association in comparison to collapsing by gene regions, as 23 gene-based results survived the correction for multiple testing (P<2.0 x 10^−4^ (250 tests)) in comparison to 47 domain-based results (P<5.0 x 10^−5^ (1,000 tests)). The tops hits from these analyses can be found in S1 File. This was due to analysing smaller blocks of variants which consisted of a higher proportion of disease causing variants (i.e. a smaller proportion of neutral variants were involved in these analyses and therefore incorporated less statistical noise). It was therefore hypothesised that, if a sufficient proportion of causal variants resided within domain regions, that our planned analyses should identify associational signals which would not be detected using a gene-based approach.

### Sample Characteristics

5,330,943 sites were excluded from further analyses due to showing Sanger/BGI batch effects, failed the test for Hardy-Weinberg equilibrium (P<1x10^-6^) or were below the VQSLOD score cut-off (Variant Quality Score Recalibration) that corresponds to the maximum truth sensitivity tranche of 99.5% compared to HapMap3.3. Filtering to only include variants with a CADD C-Score ≥ 15 reduced the final number to 546,334.

After removing samples that failed QC, we were left with a sample size of 3,621 which did not include related pairs or non-European individuals. Subsequently removing individuals with missing phenotype information resulted in a final sample size of ~3,200 (3,210 for HDL, 3,191 for LDL, 3,206 for TC and 3,202 for TG).

### Individual Domain Analysis

We mapped 76,391 protein families and domains back onto the human genome. 39,016 of which were canonical and used for all analyses. After filtering to only include variants with a CADD C-Score ≥ 15, ~9,570 domains contained at least 2 variants after applying a MAF cutoff of 1%. These varied very slightly due to the small differences in sample size after matching on phenotypes. Our threshold for multiple comparisons were therefore 5.22 x 10^−6^. No results from this analysis survived this threshold using either the SKAT or SKAT-O tests (S1 File).

### Domain-Domain Interaction Analysis

Using the iPfam and STRINGdb databases we predicted there to be 14,046 combined regions that were responsible for domain-domain interactions. After filtering to include SNVs with a CADD C-Score ≥ 15, there were ~10,020 using the 1% MAF cutoff. This determined that our threshold for multiple comparisons was 4.99 x 10^−6^. However, no results from the analysis survived this threshold (S1 File).

### Variants Collapsed by Protein Family Analysis

2,356 unique Pfam identifiers were used in the previous analyses. We collapsed all variants together within regions with the same Pfam identifier and then stratified them to only include SNPs with a CADD C-Score ≥ 15. There were 3,114 regions with 2 or more variants in after filtering using the MAF cutoff of 1%, meaning our thresholds for multiple comparisons was 1.61 x 10^−5^. We analysed these regions as before and did not observe any p-values lower than the thresholds for multiple comparisons (S1 File).

Single variant analyses were undertaken for all variant analysed previously, to ensure that collapsing variants together did not result in any association signals becoming undetectable when using this approach. The results were plotted using Q-Q plots, which did not suggest that strong evidence of association was detected using single variant analyses for each lipid trait (S1 File).

### Comparison between Individual Domain and Gene-based Results

**Fig 2:**
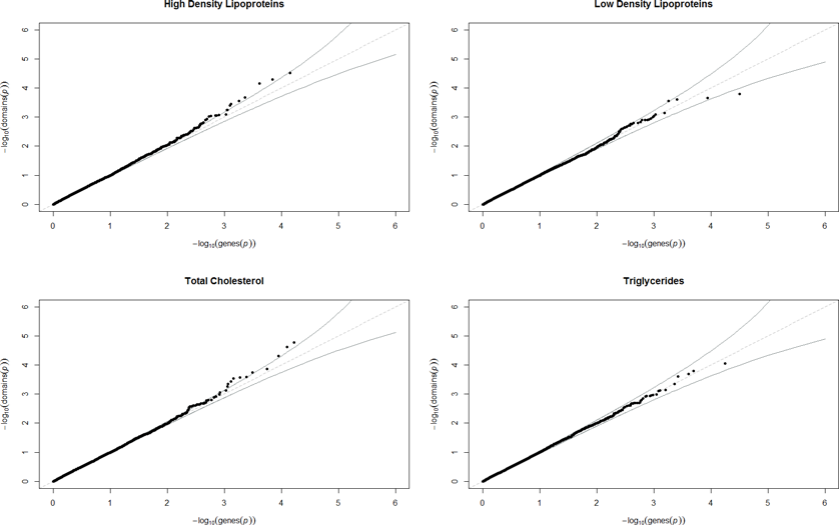
Quantile-Quantile plots to compare distributions of p-values identified using individual domain and gene-based approaches to rare variant analysis. These Q-Q plots represent the distribution of p-values from an analysis where rare variants have been collapsed together using protein domain coordinates and analysed with lipid traits. The reference distribution for these plots are distributions of p-values from an identical analysis except collapsing rare variants using conventional gene-based coordinates.

Q-Q plots which compare the distribution of p-values from the individual domain and gene-based analyses were generated. Results varied according to lipid trait, as the HDL and TC analyses suggested that the individual domain approach provided stronger evidence of association due to an uptick in signal. In contrast, the LDL and TG plots are predominantly confined within the 95% confidence intervals. These plots can be found in Fig 2.

## Discussion

We have undertaken a novel approach to rare variant analysis which, to our knowledge, is the first of its kind. Detecting strong evidence of association from multiple rare variants over an entire gene region is challenging for many reasons, not least of all the possibility that these variants may reside in different types of structural and functional domains. By aggregating variants together across protein domains and families, we hypothesised that sets of variants may be more likely to have a similar functional impact, as well as contain fewer neutral variants, than when collapsing variants across entire genes. However, we have been unable to provide evidence to support this in our study. Future studies should therefore contemplate applying this approach to large-scale sequence data to further evaluate whether collapsing variants in this manner may identify association signals not detected using gene-based approaches.

It has become common practice for studies to undertake collapsing approaches using entire gene regions, although typically their findings have been underwhelming. This is in no small part due to a large proportion of neutral variants in collapsed regions which incorporate statistical noise into the analysis. Furthermore, regarding genes as functional units can have limitations. For instance, the protein product of a gene may contain multiple domains which can be recombined in a different order and alter its overall function, which consequently can cause a different phenotypic effect downstream[30]. The study of protein domains has previously revealed important functional insights, such as the identification of *LRRK2* as a promising therapeutic target for the treatment of Parkinson’s disease[31]. Moreover, there have been studies which have shown both Mendelian disease and somatic cancer mutations to cluster within certain types of protein domains[32, 33]. Proteins have evolved through the shuffling of functional domains, causing some domain sequences to be located many times across the proteome. Protein-protein interfaces are typically more conserved than the rest of the protein surface which is why in this study we examined the combined effect of rare variants that were responsible for domain regions predicted to interact with one and other.

A limitation to using protein domain regions when aggregating rare variants together is that the extent in which protein domains are categorised varies significantly. A subset of protein domains have been thoroughly investigated, whereas the functional role of the majority of domains remains unknown. Pfam definitions are based on amino acids sequences which are repeated across the proteome according to hidden Markov models, rather than any evidence implicating them in the aetiology of complex disease. However, as future research continues to develop our understanding of the functional task of protein domains, as well as our overall understanding of the genetic architecture of complex disease, there will be additional value in aggregating variants in the manner undertaken in this study.

In terms of how we can proceed by analysing rare variants in this manner, there are valuable resources that can aid domain-centric analyses. Along with the Pfam database used here, the Conserved Domains Database (CDD)[10], the SUPERFAMILY[34] database and the Protein Analysis Through Evolutionary Relationships (PANTER)[35] resource contain a wealth of information which can be utilised to aid rare variant association analyses. Furthermore, we have used STRINGdb in this study to define PPIs as it allowed us to filter only those interactions with experimental evidence and high confidence (i.e. a STRING Score of ≥ 0.8). However, resources such as IntAct[36] contain experimentally verified binding sites and could therefore be incorporated into the analysis pipeline presented in this study. A catalogue of mutation pathogenicity prediction tools have been developed in recent years to prioritise or weigh SNPs in association studies and many of these tools use conservation score as a key variable in their predictions. In this study we have used annotations from CADD, although prediction tools such as FATHMM-MKL[37] and DANN[38] may also be useful for variant filtering.

Despite a lack of statistically robust findings in our analyses, there may still be value in examining association signals from rare variants collapsed across highly conserved regions. Moreover, the parameters and resources we have used in this study for the analysis pipeline can be adjusted and this may lead to stronger evidence of association than observed here. For instance, the resources used to define our protein domain regions, the tool used to quantify the predicted deleterious impact of variants (as well as the threshold applied to filter) and the collapsing method used to analyse genotype-phenotype associations are all variables which can be adjusted for in future studies.

In this study, we have focused on lipid traits due to the success reported by other studies in recent years [39–41]. Although these traits are typically observed to be polygenic in nature [42], there are also monogenic diseases which can caused by extreme lipid levels, such as familial hypercholesterolemia[43]. Mutations in genes such as *LDLR*, *APOB*, *PCSK9* and *LDLRAP1* are known to lead to this condition [44], although endeavours in rare variant analysis hope to underpin novel loci which harbour causal variants in lipid related diseases. However, it is also expected that rare variants may be causal to rarer diseases and thus the approach used in this study may be useful for future studies which wish to investigate this. Moreover, the studies which have had success in detecting novel loci in disease using rare variant approaches have used large sample sizes (i.e. Surakka et al had a sample size of over 60,000 individuals), in comparison to the 3,200 individuals analysed in this study. Applying the approach outlined in this study to similarly large sample sizes may therefore yield improved results. The use of the software RAREMETAL[45] could be incorporated into the framework presented in this study to facilitate analyses using samples which include multiple cohorts.

Previous studies have suggested that larger sample sizes and alternative statistical methodology should help improve findings for collapsing methods. Using a more discrete definition of a functional unit across the genome, such as protein domains and families, provides a feasible alternative to collapsing by gene coordinates, which may yield biologically meaningful inferences and previously unidentified association signals when undertaking rare variant analyses.

## Supporting Information

S1 File Supporting Information. Supplementary Material.
(DOCX)
