# Supplementary material for: A Protein Domain and Family Based Approach to Rare Variant Association Analysis
Source: PLoS One. 2016 Apr 29;11(4):e0153803. doi: 10.1371/journal.pone.0153803 (PMC4851355; doi:10.1371/journal.pone.0153803)
Supplement: S1 File — Supplementary Material. (DOCX) [file pone.0153803.s001.docx]

**Supplementary material**

*Trait Standardization:* Prior to analysis, each trait was standardized by separating males and females into separate groups. The TwinsUK participants were then further divided into two unrelated subsets. Outliers which deviated by more than 4 or 5 standard deviations from the sample mean (ALSPAC and TwinsUK respectively) for each trait were removed from analysis. All traits were inverse normal transformed within each group separately and residuals were computed by adjusting for age (as well as age^2^ for TwinsUK individuals). Furthermore, analyser effects were included as a random effect in associated with the phenotype in TwinsUK individuals. Lastly, residuals were standardized before combining groups.

**Table 1: Top 10 Gene-based results using Simulated Data**

| **Gene** | **P-value** |
| --- | --- |
| Gene228 | 1.26 x 10^-6^ |
| Gene145 | 3.23 x 10^-6^ |
| Gene133 | 3.40 x 10^-6^ |
| Gene231 | 5.36 x 10^-6^ |
| Gene156 | 5.94 x 10^-6^ |
| Gene163 | 7.47 x 10^-6^ |
| Gene84 | 9.58 x 10^-6^ |
| Gene178 | 1.06 x 10^-5^ |
| Gene189 | 1.59 x 10^-5^ |
| Gene56 | 2.03 x 10^-5^ |

P-value = P-value according to SKAT

**Table 2: Top 10 Domain-based results using Simulated Data**

| **Domain** | **P-value** |
| --- | --- |
| Domain313 | 4.31 x 10^-9^ |
| Domain255 | 6.90 x 10^-9^ |
| Domain911 | 1.44 x 10^-8^ |
| Domain657 | 2.13 x 10^-8^ |
| Domain210 | 2.47 x 10^-8^ |
| Domain442 | 4.98 x 10^-8^ |
| Domain117 | 5.14 x 10^-8^ |
| Domain110 | 5.32 x 10^-8^ |
| Domain872 | 5.87 x 10^-8^ |
| Domain45 | 6.32 x 10^-8^ |

P-value = P-value according to SKAT

Please note that the Simulated Gene/Domain IDs have no connection (i.e. Domain1 does not necessarily reside in Gene1). Regions in both analyses were randomly selected and therefore allocated an ID at random.

**SKAT results**

**Table 3: Individual Domain analysis with High Density Lipoproteins using a MAF cutoff of 1%**

| **Gene** | **Pfam ID** | **Coordinates** | **No. of variants** | **P-value** |
| --- | --- | --- | --- | --- |
| *ZNF26* | PF13465 | chr12:133587867..133587940 | 2 | 3.04 x 10^-5^ |
| *IGF2BP3* | PF14259 | chr7:23508132..23509719 | 2 | 5.05 x 10^-5^ |
| *MEF2B* | PF00319 | chr19:19260118..19261516 | 2 | 6.92 x 10^-5^ |
| *ZNF75D* | PF13465 | chrX:134421230..134421289 | 7 | 0.000276 |
| *LRP1* | PF00057 | chr12:57593788..57594317 | 2 | 0.000345 |
| *ZNF213* | PF02023 | chr16:3187399..3187676 | 2 | 0.000559 |
| *ACTN1* | PF00307 | chr14:69369211..69376743 | 9 | 0.000814 |
| *ZNF883* | PF13465 | chr9:115759714..115759787 | 7 | 0.000848 |
| *CFB* | PF00089 | chr6:31917865..31919738 | 2 | 0.000865 |
| *EIF4E* | PF01652 | chr4:99802235..99823039 | 11 | 0.000891 |

**Table 4: Individual Domain analysis with Low Density Lipoproteins using a MAF cutoff of 1%**

| **Gene** | **Pfam ID** | **Coordinates** | **No. of variants** | **P-value** |
| --- | --- | --- | --- | --- |
| *ATP2A1* | PF00702 | chr16:28900215..28913224 | 7 | 0.00016 |
| *SHOX2* | PF00046 | chr3:157818067..157820599 | 3 | 0.000216 |
| *IGFALS* | PF01462 | chr16:1842088..1842186 | 3 | 0.000244 |
| *DYRK1A* | PF00069 | chr21:38853087..38877783 | 2 | 0.000281 |
| *TMEM240* | PF15207 | chr1:1470744..1475724 | 6 | 0.000726 |
| *HP1BP3* | PF00538 | chr1:21091886..21094126 | 4 | 0.000797 |
| *ZNF17* | PF13465 | chr19:57932229..57932299 | 10 | 0.000907 |
| *DLG1* | PF00595 | chr3:196865219..196876639 | 5 | 0.000987 |
| *ZNF729* | PF13465 | chr19:22497051..22497125 | 3 | 0.001113 |
| *MCF2* | PF00621 | chrX:138680591..138692481 | 3 | 0.001203 |

**Table 5: Individual Domain analysis with Total Cholesterol using a MAF cutoff of 1%**

| **Gene** | **Pfam ID** | **Coordinates** | **No. of variants** | **P-value** |
| --- | --- | --- | --- | --- |
| *BOP1* | PF00400 | chr8:145486374..145486559 | 2 | 1.67 x 10^-5^ |
| *RGS6* | PF00610 | chr14:72818845..72925082 | 25 | 2.35 x 10^-5^ |
| *FN1* | PF00039 | chr2:216298059..216298171 | 4 | 4.82 x 10^-5^ |
| *HP1BP3* | PF00538 | chr1:21091886..21094126 | 4 | 0.000135 |
| *WDR47* | PF00400 | chr1:109517170..109517267 | 3 | 0.000255 |
| *DYRK1A* | PF00069 | chr21:38853087..38877783 | 2 | 0.000271 |
| *ANK1* | PF12796 | chr8:41581064..41583361 | 3 | 0.000283 |
| *TMTC4* | PF08409 | chr13:101288829..101289864 | 3 | 0.000365 |
| *LAMC2* | PF00053 | chr1:183197589..183197750 | 2 | 0.000441 |
| *QSOX1* | PF04777 | chr1:180159640..180165439 | 4 | 0.000546 |

**Table 6: Individual Domain analysis with Triglycerides using a MAF cutoff of 1%**

| **Gene** | **Pfam ID** | **Coordinates** | **No. of variants** | **P-value** |
| --- | --- | --- | --- | --- |
| *ADRBK1* | PF00169 | chr11:67052338..67052800 | 4 | 7.04 x 10^-5^ |
| *CYP17A1* | PF00067 | chr10:104590512..104597036 | 2 | 8.72 x 10^-5^ |
| *NCOA4* | PF12489 | chr10:51579250..51582203 | 2 | 0.000156 |
| *CR1* | PF00084 | chr1:207715535..207715735 | 4 | 0.000204 |
| *NEDD9* | PF14604 | chr6:11213795..11213944 | 6 | 0.000242 |
| *IGF2BP3* | PF00013 | chr7:23352064..23353199 | 4 | 0.000452 |
| *RGS9* | PF00610 | chr17:63149579..63156454 | 2 | 0.000722 |
| *MSH5* | PF05192 | chr6:31713057..31727725 | 4 | 0.00075 |
| *KLK3* | PF00089 | chr19:51359522..51361850 | 3 | 0.00078 |
| *NRG1* | PF07679 | chr8:32453366..32463188 | 2 | 0.000782 |

**SKAT-O results**

**Table 7: Individual Domain analysis with High Density Lipoproteins using a MAF cutoff of 1%**

| **Gene** | **Pfam ID** | **Coordinates** | **No. of variants** | **P-value** |
| --- | --- | --- | --- | --- |
| *ANK3* | PF00791 | chr10:61865738..61873986 | 2 | 3.24 x 10^-5^ |
| *HGS* | PF12210 | chr17:79662852..79663496 | 3 | 6.27 x 10^-5^ |
| *CA8* | PF00194 | chr8:61121352..61193620 | 2 | 9.16 x 10^-5^ |
| *LRP1* | PF00057 | chr12:57593788..57594317 | 2 | 0.000369 |
| *LRRC37A* | PF14914 | chr17:44409030..44412946 | 2 | 0.000392 |
| *ZNF213* | PF02023 | chr16:3187399..3187676 | 2 | 0.000413 |
| *ATP2B1* | PF00690 | chr12:90035980..90049508 | 4 | 0.000556 |
| *BRCA1* | PF12820 | chr17:41246029..41246517 | 8 | 0.000586 |
| *CALU* | PF13833 | chr7:128407689..128409169 | 2 | 0.00064 |
| *ZNF883* | PF13465 | chr9:115759714..115759787 | 7 | 0.000726 |

**Table 8: Individual Domain analysis with Low Density Lipoproteins using a MAF cutoff of 1%**

| **Gene** | **Pfam ID** | **Coordinates** | **No. of variants** | **P-value** |
| --- | --- | --- | --- | --- |
| *BOP1* | PF00400 | chr8:145486374..145486559 | 2 | 5.69 x 10^-5^ |
| *ALPP* | PF00245 | chr2:233243767..233246366 | 3 | 0.000168 |
| *FUCA1* | PF01120 | chr1:24175188..24194721 | 2 | 0.000231 |
| *ADCY9* | PF00211 | chr16:4016114..4016688 | 3 | 0.000265 |
| *LIPK* | PF00561 | chr10:90490847..90512465 | 2 | 0.000444 |
| *DCC* | PF00041 | chr18:50683752..50705454 | 3 | 0.000899 |
| *MLLT3* | PF03366 | chr9:20448210..20620760 | 4 | 0.001023 |
| *LAMA2* | PF00053 | chr6:129634009..129634155 | 2 | 0.001064 |
| *ALB* | PF13405 | chr16:71418707..71419527 | 2 | 0.001234 |
| *SESN3* | PF04636 | chr11:94917617..94926651 | 2 | 0.0016 |

**Table 9: Individual Domain analysis with Total Cholesterol using a MAF cutoff of 1%**

| **Gene** | **Pfam ID** | **Coordinates** | **No. of variants** | **P-value** |
| --- | --- | --- | --- | --- |
| *BOP1* | PF00400 | chr8:145486374..145486559 | 2 | 1.65 x 10^-5^ |
| *FN1* | PF00039 | chr2:216298059..216298171 | 4 | 4.73 x 10^-5^ |
| *RGS6* | PF00610 | chr14:72818845..72925082 | 25 | 5.10 x 10^-5^ |
| *DCC* | PF00041 | chr18:50683752..50705454 | 3 | 6.20 x 10^-5^ |
| *ALPP* | PF00245 | chr2:233243767..233246366 | 3 | 0.00011 |
| *MX1* | PF00350 | chr21:42807875..42813656 | 2 | 0.000265 |
| *MEF2C* | PF00319 | chr5:88100498..88119576 | 2 | 0.000303 |
| *BRPF1* | PF00439 | chr3:9786763..9787613 | 4 | 0.000455 |
| *HOXC13* | PF00046 | chr12:54338828..54338998 | 8 | 0.000498 |
| *FUCA1* | PF01120 | chr1:24175188..24194721 | 2 | 0.000832 |

**Table 10: Individual Domain analysis with Triglycerides using a MAF cutoff of 1%**

| **Gene** | **Pfam ID** | **Coordinates** | **No. of variants** | **P-value** |
| --- | --- | --- | --- | --- |
| *ADRBK1* | PF00169 | chr11:67052338..67052800 | 4 | 8.06 x 10^-5^ |
| *CYP17A1* | PF00067 | chr10:104590512..104597036 | 2 | 8.90 x 10^-5^ |
| *NCOA4* | PF12489 | chr10:51579250..51582203 | 2 | 0.000128 |
| *ENPP7* | PF01223 | chr6:132201103..132211579 | 2 | 0.000211 |
| *IFIT5* | PF00515 | chr10:91178265..91178360 | 5 | 0.000368 |
| *BRAF* | PF00130 | chr7:140501234..140507766 | 2 | 0.00077 |
| *SEC14L2* | PF00650 | chr22:30803404..30811814 | 2 | 0.000771 |
| *RGS9* | PF00610 | chr17:63149579..63156454 | 2 | 0.000782 |
| *PPP1R12A* | PF12796 | chr12:80239169..80328575 | 2 | 0.000846 |
| *NRG1* | PF07679 | chr8:32453366..32463188 | 2 | 0.000894 |

**Table 11: Domain-Domain Interaction Combined Variant Analysis with HDL - MAF 1%**

| **Gene A** | **Domain A** | **Gene B** | **Domain B** | **No. of Variants** | **P-value** |
| --- | --- | --- | --- | --- | --- |
| *IRF8* | PF00605 | *IRF6* | PF00605 | 10 | 0.000113 |
| *IRS2* | PF00169 | *PIK3R1* | PF00620 | 9 | 0.000389 |
| *SIK1* | PF00069 | *STK11* | PF00069 | 12 | 0.000715 |
| *IRS2* | PF00169 | *GRB10* | PF00788 | 8 | 0.000725 |
| *IRS1* | PF00169 | *ROCK1* | PF00169 | 10 | 0.000776 |
| *IRS2* | PF00169 | *PIK3R2* | PF00620 | 7 | 0.000848 |
| *ISL1* | PF00046 | *LHX4* | PF00046 | 8 | 0.000852 |
| *SIGLEC11* | PF00047 | *PTPN6* | PF00102 | 11 | 0.000891 |
| *SIK2* | PF00069 | *PPP2CA* | PF00149 | 11 | 0.000891 |
| *SHOX2* | PF00046 | *ELAVL1* | PF00076 | 13 | 0.000922 |

**Table 12: Domain-Domain Interaction Combined Variant Analysis with LDL - MAF 1%**

| **Gene A** | **Domain A** | **Gene B** | **Domain B** | **No. of Variants** | **P-value** |
| --- | --- | --- | --- | --- | --- |
| *CLK2* | PF00069 | *TGFBR1* | PF00069 | 4 | 0.001705 |
| *EIF2AK1* | PF00069 | *EIF2AK2* | PF00069 | 4 | 0.001796 |
| *STAT3* | PF00017 | *STAT6* | PF00017 | 3 | 0.001971 |
| *DNM2* | PF00169 | *VAV1* | PF00169 | 12 | 0.002306 |
| *SH3BP2* | PF00017 | *GRB2* | PF00018 | 3 | 0.002474 |
| *SH3BP2* | PF00017 | *LCK* | PF00018 | 4 | 0.003292 |
| *MSH6* | PF01624 | *MSH2* | PF01624 | 2 | 0.003904 |
| *CLNK* | PF00017 | *LCP2* | PF00017 | 17 | 0.004627 |
| *ABL1* | PF07714 | *JAK1* | PF07714 | 2 | 0.005435 |
| *HCK* | PF00017 | *BCR* | PF00169 | 12 | 0.005872 |

**Table 13: Domain-Domain Interaction Combined Variant Analysis with TC - MAF 1%**

| **Gene A** | **Domain A** | **Gene B** | **Domain B** | **No. of Variants** | **P-value** |
| --- | --- | --- | --- | --- | --- |
| *BTK* | PF00018 | *KIT* | PF00047 | 2 | 1.67 x 10^-5^ |
| *EIF2AK1* | PF00069 | *EIF2AK2* | PF00069 | 4 | 0.000256 |
| *CLK2* | PF00069 | *TGFBR1* | PF00069 | 4 | 0.000546 |
| *MSH6* | PF01624 | *MSH2* | PF01624 | 2 | 0.0006 |
| *CRK* | PF00018 | *PIK3R1* | PF00620 | 6 | 0.001357 |
| *CYP11A1* | PF00067 | *CYP11B2* | PF00067 | 23 | 0.001596 |
| *BMPR2* | PF00069 | *MOS* | PF00069 | 4 | 0.001601 |
| *RASA1* | PF00169 | *BCR* | PF00621 | 18 | 0.001835 |
| *CNTNAP2* | PF00008 | *CNTN2* | PF00041 | 15 | 0.002192 |
| *NFYB* | PF00808 | *CHRAC1* | PF00808 | 16 | 0.002213 |

**Table 14: Domain-Domain Interaction Combined Variant Analysis with TG - MAF 1%**

| **Gene A** | **Domain A** | **Gene B** | **Domain B** | **No. of Variants** | **P-value** |
| --- | --- | --- | --- | --- | --- |
| *POLR3B* | PF04561 | *POLR1B* | PF04565 | 4 | 7.04 x 10^-5^ |
| *RHOA* | PF00071 | *ARHGAP20* | PF00620 | 4 | 7.59 x 10^-5^ |
| *RHEB* | PF00071 | *RAB9A* | PF00071 | 2 | 8.72 x 10^-5^ |
| *RHEB* | PF00071 | *RAB7A* | PF00071 | 4 | 0.000102 |
| *RHOA* | PF00071 | *ARHGAP21* | PF00169 | 10 | 0.000278 |
| *GRB2* | PF00017 | *SHC4* | PF00640 | 4 | 0.000582 |
| *CLEC2D* | PF00059 | *KLRB1* | PF00059 | 2 | 0.000722 |
| *CRKL* | PF00018 | *BCAR1* | PF00018 | 5 | 0.000753 |
| *GRB2* | PF00017 | *ARHGEF7* | PF00169 | 3 | 0.000754 |
| *ITSN2* | PF00018 | *FCHSD2* | PF00018 | 3 | 0.000768 |

**Table 15: Protein Family Combined Variant Analysis according to Pfam ID with HDL using a MAF cutoff of 1%**

| **Pfam ID** | **No. of Variants** | **P-value** |
| --- | --- | --- |
| PF00780 | 3 | 9.38 x 10^-5^ |
| PF11936 | 5 | 0.000354 |
| PF02493 | 6 | 0.000473 |
| PF02874 | 2 | 0.000763 |
| PF01920 | 9 | 0.001461 |
| PF13793 | 2 | 0.002991 |
| PF01437 | 3 | 0.003189 |
| PF02782 | 4 | 0.003669 |
| PF09110 | 2 | 0.004179 |
| PF01722 | 5 | 0.004252 |

**Table 16: Protein Family Combined Variant Analysis according to Pfam ID with LDL using a MAF cutoff of 1%**

| **Pfam ID** | **No. of Variants** | **P-value** |
| --- | --- | --- |
| PF2714 | 2 | 0.000133 |
| PF02460 | 5 | 0.000247 |
| PF05773 | 8 | 0.000711 |
| PF14843 | 7 | 0.001368 |
| PF007565 | 13 | 0.00328 |
| PF00240 | 4 | 0.003297 |
| PF05383 | 5 | 0.004113 |
| PF00249 | 3 | 0.00447 |
| PF15365 | 4 | 0.005676 |
| PF01590 | 11 | 0.005988 |

**Table 17: Protein Family Combined Variant Analysis according to Pfam ID with TC using a MAF cutoff of 1%**

| **Pfam ID** | **No. of Variants** | **P-value** |
| --- | --- | --- |
| PF13639 | 7 | 5.73 x 10^-5^ |
| PF00989 | 3 | 0.000187 |
| PF11881 | 5 | 0.000313 |
| PF03184 | 6 | 0.00087 |
| PF03637 | 5 | 0.000989 |
| PF06009 | 2 | 0.001134 |
| PF00341 | 7 | 0.001787 |
| PF04738 | 2 | 0.002113 |
| PF00188 | 4 | 0.002138 |
| PF00168 | 11 | 0.00431 |

**Table 18: Protein Family Combined Variant Analysis according to Pfam ID with TG using a MAF cutoff of 1%**

| **Pfam ID** | **No. of Variants** | **P-value** |
| --- | --- | --- |
| PF10582 | 8 | 0.000212 |
| PF02932 | 3 | 0.001338 |
| PF02064 | 4 | 0.00337 |
| PF00412 | 28 | 0.003677 |
| PF09799 | 9 | 0.00444 |
| PF12409 | 7 | 0.004476 |
| PF13831 | 3 | 0.004651 |
| PF01825 | 9 | 0.004317 |
| PF07525 | 4 | 0.005663 |
| PF00200 | 7 | 0.006109 |

**Supplementary Figure 1: Quantile-Quantile plots for single variant analyses with 4 lipid traits**

**
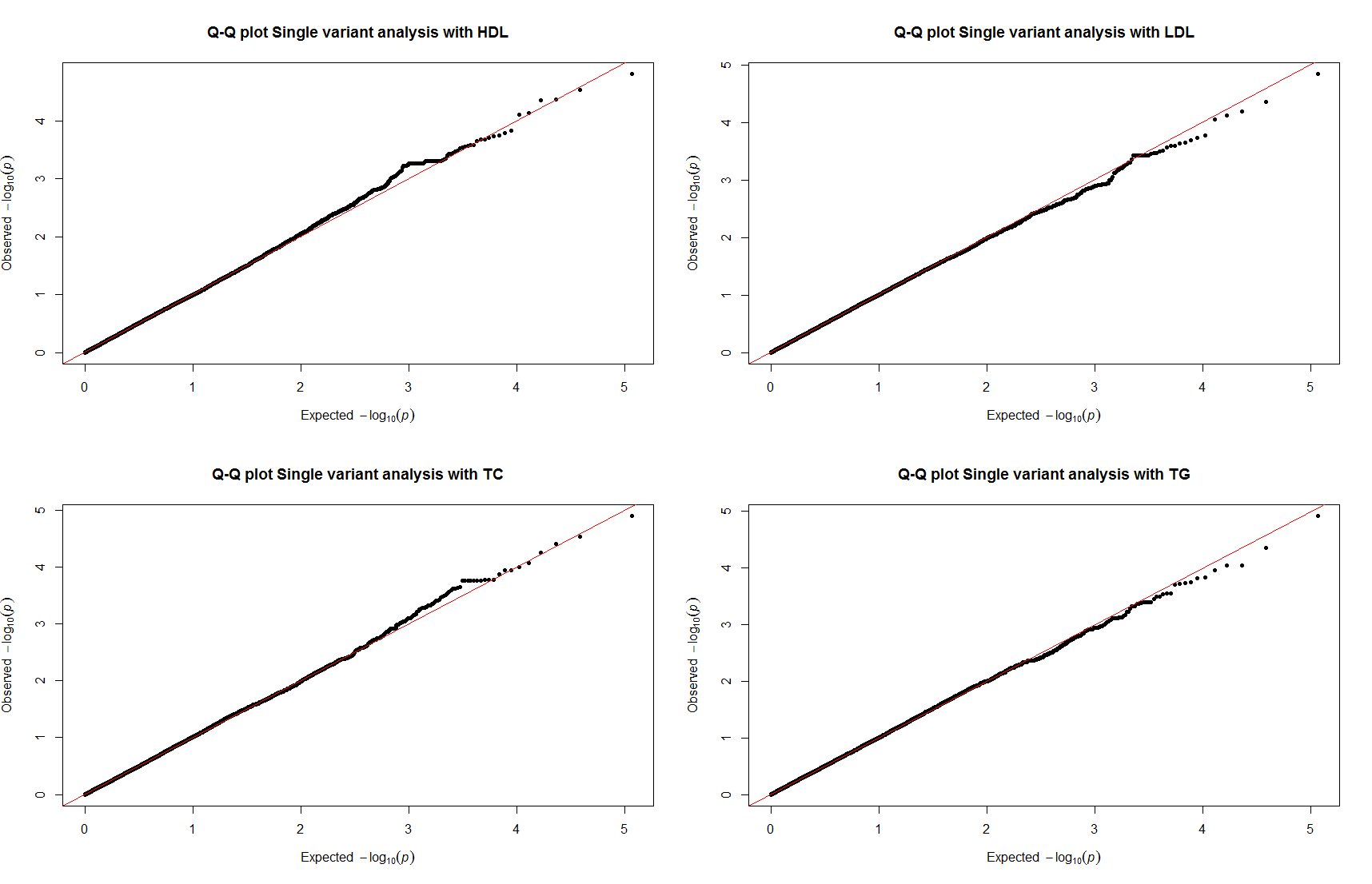
**
